# Misreporting and misclassification: implications for socioeconomic disparities in body-mass index and obesity

**DOI:** 10.1007/s10198-013-0545-5

**Published:** 2013-12-21

**Authors:** Åsa Ljungvall, Ulf G. Gerdtham, Ulf Lindblad

**Affiliations:** 1 Department of Economics, Lund University, P.O. Box 7082, 220 07 Lund, Sweden; 2 Health Economics and Management, Lund University, Lund, Sweden; 3 Centre for Primary Health Care Research, Lund University, Lund, Sweden; 4 Department of Public Health and Community Medicine, University of Gothenburg, Gothenburg, Sweden

**Keywords:** Misreporting, Misclassification, BMI, Obesity, Waist circumference, Education, Income, C18, I12, I14

## Abstract

Body-mass index (BMI) has become the standard proxy for obesity in social science research. This study deals with the potential problems related to, first, relying on self-reported weight and height to calculate BMI (*misreporting*), and, second, the concern that BMI is a deficient measure of body fat (*misclassification*). Using a regional Swedish sample, we analyze whether socioeconomic disparities in BMI are biased because of misreporting, and whether socioeconomic disparities in the risk of obesity are sensitive to whether BMI or waist circumference is used to define obesity. Education and income are used as socioeconomic indicators. The overall conclusion is that misreporting and misclassification may indeed matter for estimated educational and income disparities in BMI and obesity. In the misreporting part we find that women with higher education misreport less than those with lower education, leading to underestimation of the education disparity when using self-reported information. In the misclassification part we find that the probability of being misclassified decreases with income, for both men and women. Among women, the consequence is a steeper income gradient when obesity is defined using waist circumference instead of BMI. Among men the income gradient is statistically insignificant irrespective of how obesity is defined, but when estimating the probability of obesity defined by waist circumference, an educational gradient, which is not present when classifying men using BMI, arises.

## Introduction

Obesity is nowadays recognized as an important public health concern, and considerable obesity-related research is being produced in different fields. A common feature in most of the obesity research in social sciences is that, despite its shortcomings, body-mass index (BMI, calculated as weight in kilos divided by height in meters squared, kg/m^2^) has become the standard proxy for body fat and is the most widely used indicator for obesity, where obesity is defined as BMI ≥ 30 [1, 2]. BMI, calculated from self-reported, or objectively measured, weight and height, is often the only body measure available. It has the important advantage of being relatively easy and cheap to collect, especially if weight and height are self-reported. However, many studies indicate that the self-reported weight and height are misreported in a way that tends to understate BMI [3].

There is also some evidence that misreporting may vary systematically across socioeconomic groups. Nyholm et al. [4] use a Swedish regional data set (partly the same as is used in the current study) and report that there is a slight tendency for men in the middle, and women in the highest, educational group to report more accurate values of weight and height. Using another Swedish regional data set, collected in 1984–1985, Boström and Diderichsen [5] find some evidence for differences in misreporting by occupation. Dekkers et al. [6] find that misreporting is smaller in the higher educational group in a sample of overweight employees in the Netherlands. However, controlling for a broad set of covariates, Gil and Mora [7] find no systematic differences across education and individual deprivation in the misreporting of weight or height in a Spanish data set.

Clearly, misreporting of BMI could also mean that obesity rates are underestimated when defining obesity based on BMI values and using self-reported BMI to classify people. O’Neill and Sweetman [8] consider this issue and estimate upper and lower bounds for prevalence of obesity in ten European countries. To get accurate estimates of the prevalence of obesity, Madden [9] suggests lowering the threshold for the obesity definition when basing the estimate on self-reported data.

In statistical terms, misreporting is an example of measurement error, which may introduce bias in the estimated parameters in a regression where BMI is used either as a dependent or an independent variable. The direction and severity of the bias depends on the model specification and how the measurement error is related to all other variables in the model [10]. In theory, one way to overcome the measurement error problem is to use an external data set with more accurate data to quantify the error and thereafter correct for it in the primary data set [10]. In a study on the relationship between wages and obesity, Cawley [11] uses such a strategy to correct self-reported weight and height in a US data set. The relationship between the self-reported and measured information in the validation data is used to adjust the self-reported data in the primary data set, and these adjusted values are used in the analysis, instead of the original self-reported values. Several other studies, using US data sets with only self-reported height and weight, follow this method [12–17]. Gil and Mora [7] and Mora and Gil [18] apply the same method to Spanish data, and Hajizadeh et al. [19] use a similar correction procedure for Canadian data. Some of these studies explicitly note that the choice between self-reported or adjusted BMI does not affect the results substantially [11, 12, 16].

It is unclear whether the method to adjust self-reported BMI by the use of external data and prediction equations eliminates the measurement error and bias. Plankey et al. [20] find that there are measurement errors also in the adjusted measure of BMI, and that these are correlated with true BMI, and hence errors are not eliminated. Modeling income as a function of BMI among women, O’Neill and Sweetman [21] show that the effect of BMI on income is overstated when BMI is calculated from self-reports and, importantly, adjustments with auxiliary data only marginally reduces this bias. These results may explain why authors note that results are similar irrespective of whether the original self-reported or the adjusted values of BMI are used in the analysis.

Hence, misreported BMI is acknowledged in the literature. Some studies adjust their analyses by the use of auxiliary data, and there are studies aiming at estimating accurate obesity rates from self-reported data. Our study distinguishes itself from the previous by using Swedish data and by not aiming at correcting measurement error in any primary data set, but instead focusing explicitly on the misreporting behavior per se, with the aim of analyzing how socioeconomic disparities are affected. Particular attention is paid to income and education. Unlike O’Neill and Sweetman [21], who take a labor economics approach and model income as a function of BMI, we take the reverse approach and model BMI and obesity as a function of socioeconomic variables. The analysis illustrates whether and how the use of self-reported weight and height to calculate BMI, and the common use of BMI to define obesity, matter for socioeconomic disparities.

The analysis is based on regional Swedish data and consists of two parts. The first deals with *misreporting* in BMI, with the specific purpose of analyzing whether misreporting behavior varies systematically across socioeconomic groups. If it does, socioeconomic gradients based on self-reported data will be biased. We find that among women there are significant differences in reporting behavior across education, leading to underestimation of educational disparities in BMI when using self-reported information. Among men, we find no evidence of systematic differences across socioeconomic groups.

The second part deals with *misclassification* and goes beyond the standard definition of obesity as BMI ≥ 30. In US data, obesity prevalence is much higher when defining obesity based on alternative measures of body fat (estimated from bioelectrical impedance analysis [1] and skinfold thickness [22]) instead of BMI. Moreover, the negative correlation between employment and obesity increases for men, but not for women, when using the alternative measure of obesity [1]. In this study, we use waist circumference as an indicator of *abdominal* obesity, and as an alternative measure of elevated health risk. Central obesity is considered to provide an independent prediction of risk beyond BMI, in particular among individuals with BMI < 35 [2]. Unlike BMI, waist circumference takes fat distribution into account. High-risk central obesity is defined as a waist circumference of more than 88 cm for women, and more than 102 cm for men [2, 23]. We use these cut-off points, together with the standard definition of obesity as BMI ≥ 30, to explore whether *misclassification*, defined as being classified as obese according to the waist circumference definition but not according to the BMI definition, is systematically related to socioeconomic status.

As a summarizing step, we finally estimate socioeconomic gradients in obesity for three different definitions of obesity: waist circumference and BMI ≥ 30 calculated from self-reported and measured weight and height, respectively. For women, we find a steeper income gradient when obesity is defined using waist circumference instead of BMI. Among men, the income gradient is statistically insignificant irrespective of how obesity is defined, but when estimating the probability of obesity defined by waist circumference, an educational gradient, which is not present when classifying men using BMI, arises.

Taken together, the study contributes by shedding light on misreporting and misclassification patterns, and how income and educational disparities in BMI and obesity are affected. Whether there exists systematic misreporting and misclassification across socioeconomic groups is an important issue for a wide range of obesity research where self-reported weight and height are used, and where obesity is defined as BMI ≥ 30. The focus in this study is on socioeconomic disparities. Because many data sets contain only self-reported height and weight as body measures, and because it is important to track and explore disparities in excess weight, self-reported values and BMI are used as the best available option, and socioeconomic disparities in BMI and/or obesity are analyzed based on these values [24–30]. Our study explains and shows how the systematic misreporting behavior affects socioeconomic disparities in BMI, and how the misclassification affects disparities in the risk of obesity. The same patterns may hold for other populations as well. For example, a finding of a downward bias in the educational disparity in BMI among women may mean that results regarding educational disparities in other studies are underestimated too.

## Methods

### Misreporting

In analyzing socioeconomic disparities, the relationship of interest is whether and how *actual* BMI differs across socioeconomic groups, as specified in the following linear regression framework:

1 $$ {\text{BMI}}\_{\text{meas}}_{i} = \alpha^{\text{meas}} + {\mathbf{z}}_{{\mathbf{i}}} *{\varvec{\upbeta}}^{\text{meas}} + {\mathbf{x}}_{{\mathbf{i}}} *{\varvec{\upgamma}}^{\text{meas}} + e_{i} $$

where BMI_meas_*i*_ is BMI calculated from objectively measured height and weight for individual *i*, and ***z***_***i***_ is a row vector that consists of civil status (unmarried, divorced, and widow, keeping married and cohabiting individuals as reference), immigration status (first and second generation, keeping Swedish born with both parents born in Sweden as reference), and age. To allow for flexibility in the relationship between age and BMI, we model the age effect with 46 dummy variables, one for each age between 31 and 76, keeping individuals at age 30 as reference.^1^ ***x***_***i***_ is a row vector of socioeconomic variables and *e*_*i*_ is a residual term. Finally, ***γ*** ^meas^ are the parameters of main interest and reveal whether BMI differs across socioeconomic groups, given the ***z*** vector. We define ***γ*** ^meas^ as the “true” socioeconomic gradient, with the logic that it is true in the sense that it is estimated from true BMI.

When true BMI is not available, self-reported data are used instead:

2 $$ {\text{BMI}}\_{\text{self}}_{i} = \alpha^{\text{self}} + {\mathbf{z}}_{i} *{\varvec{\upbeta}}^{\text{self}} + {\mathbf{x}}_{i} *{\varvec{\upgamma}}^{\text{self}} + \varepsilon_{i} $$

where BMI_self_*i*_ refers to BMI calculated from self-reported weight and height, and all other notation is as before. Despite the use of BMI_self_*i*_ in estimating Eq. 2, the parameters of interest are still ***γ*** ^meas^. Hence, it is relevant to ask whether ***γ*** ^meas^ = ***γ*** ^self^, and thereby whether ***γ*** ^self^ are unbiased estimates of the “true” disparities. The following equation tests whether ***γ*** ^meas^ = ***γ*** ^self^:

3 $$ {\text{BMI}}\_{\text{self}}_{i} -{\text{BMI}}\_{\text{meas}}_{i} = \alpha_{3} + {\mathbf{z}}_{{\mathbf{i}}} *{\varvec{\upbeta}}^{\text{total}} + {\mathbf{x}}_{i} *{\varvec{\upgamma}}^{\text{total}} + \tau_{i} $$

where BMI_self_*i*_ –BMI_meas_*i*_ is defined as misreporting, and other notation is as before. If ***γ*** ^total^ ≠ 0, ***γ*** ^meas^ and ***γ*** ^self^ are different, and hence there is bias in the estimated disparities based on the self-reported data.

The potential bias in ***γ*** ^self^ consists of a direct and an indirect effect of socioeconomic status. To see this, it is useful to express misreporting as a function of true BMI:

4 $$ {\text{BMI}}\_{\text{self}}_{i} {-}{\text{BMI}}\_{\text{meas}}_{i} = \alpha_{4} + \rho *{\text{BMI}}\_{\text{meas}}_{i} + {\mathbf{z}}_{{\mathbf{i}}} *{\varvec{\upbeta}}^{\text{direct}} + {\mathbf{x}}_{{\mathbf{i}}} *{\varvec{\upgamma}}^{\text{direct}} + r_{i} $$

where notation is as before.^2^ *ρ* reveals whether misreporting is related to the level of true BMI. BMI_self_*i*_ − BMI_meas_*i*_ < 0 means that BMI calculated from self-reported weight and height is underreported, and *ρ* < 0 implies that underreporting increases with the true level of BMI. If ***γ*** ^direct^ > 0, underreporting decreases with socioeconomic status, *given the same level of true BMI* and ***z***. This is referred to as the direct effect of socioeconomic status on misreporting. To see the indirect effect as well, substitute Eq. 1 into the right hand side of Eq. 4:5where notation is as before. Equation 5 shows that the total misreporting attributable to the socioeconomic status variable *x*_*k*_ can be decomposed into (*γ*_*k*_^meas^ *ρ* + *γ*_*k*_^direct^). Hence, the total difference related to socioeconomic status consists of the direct effect shown in Eq. 4, *γ*_*k*_^direct^, and an indirect effect *γ*_*k*_^meas^ *ρ*. The indirect effect is a combination of the “true” gradient in BMI, *γ*_*k*_^meas^, and the effect of measured BMI on misreporting behavior. Hence, the indirect effect appears if true BMI varies systematically with socioeconomic status, and if misreporting additionally is related to true BMI.

Previous studies that analyze misreporting in self-reported weight and height do not discuss, or distinguish between, the direct and indirect effect [4, 6, 7]. Some of them measure the total effect [4], as in Eq. 3, and some of them measure the direct effect through an approach similar to Eq. 4 [6, 7]. In this study, we consider the total difference across socioeconomic groups, as well as the decomposition into direct and indirect effects. We estimate Eqs. 1 and 2 to compare the resulting disparities when using objectively measured and self-reported data, respectively. We then estimate Eq. 3 and test the hypothesis that ***γ*** ^total^ = 0. Following Eq. 5, the potential bias in ***γ*** ^self^ can be decomposed into a direct and an indirect effect. The direct effect, ***γ*** ^direct^, is estimated in Eq. 4, while the indirect effect is estimated in Eqs. 4 (*ρ*) and 1 (***γ*** ^meas^). The equations are estimated by OLS with robust standard errors, assuming that the residual terms are normally distributed with a zero mean, and are estimated for men and women separately.^3^

Underreporting of BMI is well known, and tends to increase with the true level of BMI. We therefore expect *ρ* < 0 in Eq. 4. Regarding the direct effect of socioeconomic status, ***γ*** ^direct^ in Eq. 4, there is no straightforward theoretical argument for the direction. Higher socioeconomic status may imply more informed individuals, who keep track of the public debate on the development of, and the risks related to, obesity to a larger extent, and who could potentially therefore be aware of the development of their own body to a larger extent. This argument implies less misreporting with higher socioeconomic status, and hence ***γ*** ^direct^ > 0. On the other hand, although better informed individuals in higher socioeconomic groups could lead to more accurate reporting behavior, it may also lead to less accurate reporting, because the reported weight and height could be the desired outcomes. Knowledge of the risks related to obesity may lead to lower desired than actual BMI. Further, the ideal image may differ across socioeconomic groups, with the possibility that the norm of a fit and normal-weight body is stronger in higher socioeconomic groups. These two arguments imply increasing misreporting with socioeconomic status, i.e. ***γ*** ^direct^ < 0. Although ambiguous in theory, there is some empirical evidence showing a tendency towards ***γ*** ^direct^ > 0 [6, 31].

Finally, a common finding in the literature is that BMI decreases with socioeconomic status, and we therefore expect ***γ*** ^meas^ < 0. As can be seen from Eq. 5, these expectations together, *ρ* < 0, ***γ*** ^direct^ > 0, and ***γ*** ^meas^ < 0, imply that the socioeconomic disparity estimated from self-reported weight and height is likely to be biased towards zero.

### Misclassification

The second part of the analysis deals with misclassification, defined as having a waist circumference above the cut-off point for high risk of adverse health outcomes (88 cm for women, 102 cm for men), but not being categorized as obese based on BMI calculated from objectively measured weight and height, where obesity is defined as BMI ≥ 30 for both men and women.

The relationship between misclassification and socioeconomic status is estimated by OLS in a linear probability model, with robust standard errors:

6 $$ { \Pr }\left( {{\text{misclassified}}_{i} } \right) = \alpha_{6} + {\mathbf{z}}_{{\mathbf{i}}} *{\varvec{\upbeta}}^{\text{miss}} + {\mathbf{x}}_{{\mathbf{i}}} *{\varvec{\updelta}}^{\text{miss}} + \varepsilon_{i} $$

where notation is as before. The parameters of main interest are ***δ*** ^miss^ and indicate whether socioeconomic status is related to the probability of being misclassified.

Finally, to see directly whether different definitions of obesity result in different socioeconomic gradients, we estimate the risk of being obese as a function of age and socioeconomic status for three different definitions of obesity:

7 $$ Pr\left( {{\text{obese}}_{i} } \right) = \alpha_{7} + {\mathbf{z}}_{{\mathbf{i}}} *{\varvec{\upbeta}}^{\text{obese}} + {\mathbf{x}}_{{\mathbf{i}}} *{\varvec{\updelta}}^{\text{obese}} + \varepsilon_{i} $$

where obese_*i*_ is defined using BMI ≥ 30 based on self-reported or measured weight and height, or using waist circumference. Equation 7 is estimated by OLS with robust standard errors.

### Data, variables and sample

To date, there is no nationally representative data set that contains *measured* information about weight and height in Sweden. Our analysis therefore uses a regional sample, collected between 2001 and 2005 in a region in the south of Sweden. The data set consists of two surveys. One was conducted between 2001 and 2004 in the municipality of Vara (participation rate 81 %), and the other between 2004 and 2005 in the nearby municipality of Skövde (participation rate 70 %). In each survey, individuals aged between 30 and 76 were randomly selected from the population in strata by age and sex, and invited to make two visits to a health care center. On the first visit, participants answered a questionnaire including questions about civil status, immigration background and their height and weight. When they came back for the second visit, their height and weight were measured, which they were unaware of when they filled out the questionnaire. Waist circumference (in centimeters) was also measured at this time.^4^

We linked register data on education and income from Statistics Sweden to the survey data. Individuals are classified into four educational groups: up to 11 years of schooling (*educ1*), 2 or 3 years of high school (*educ2*), up to 3 years of university or other post-secondary education (*educ3*), and at least 3 years of post-secondary education (*educ4*). The income measure is household disposable income per consumption unit.^5^

Pregnant women (*n* = 9) are excluded from the final sample. Further, to avoid results being driven by a few outliers, the 1 % with largest (absolute) misreporting (*n* = 28) and the 1 % top and bottom income observations (*n* = 56) are excluded from the analysis. One more observation is lost due to missing information on income. Thirty-five observations lack register information on education. Twenty-nine of these have self-reported information which is used instead, and the other six observations are excluded from the analysis. Observations with missing information on waist circumference (*n* = 1), measured BMI (*n* = 2), self-reported BMI (*n* = 141), civil status (*n* = 7), and immigration status (*n* = 6) are also excluded. The final sample consists of 1,266 female and 1,294 male observations. No sample weights are provided in the data set or used in the analysis. Hence, the results are not necessarily representative for the full population that was first invited to participate in the surveys.

## Results

### Descriptive statistics

Table 1 reports descriptive statistics for the final sample. As expected, BMI calculated from measured height and weight is higher than BMI based on self-reports, and underreporting is related to the level of BMI. Stratifying by BMI classification (underweight: BMI < 18.5, normal weight: 18.5 ≤ BMI < 25, overweight: ≤25 BMI < 30, and obese: BMI ≥ 30) shows that, on average, the underweight overreport BMI, whereas both men and women in the three other BMI statuses underreport. The obese underreport more than the overweight, who in turn underreport more than normal-weight individuals.

**Table 1:** Descriptive statistics. Final sample

	Women					Men				
	*n*	Mean	SD	Min	Max	*n*	Mean	SD	Min	Max
BMI										
BMI (measured)	1,266	26.52	5.22	15.78	52.60	1,294	26.81	3.56	17.47	49.17
BMI (self-reported)	1,266	25.85	5.02	15.43	51.90	1,294	26.30	3.40	17.93	48.42
BMI (self-reported) − BMI (measured)	1,266	−0.67	0.91	−4.14	4.06	1,294	−0.51	0.92	−3.66	3.19
BMI_self − BMI_meas if BMI_meas < 18.5	11	0.44	0.65	−0.35	1.80	1	0.45		0.45	0.45
BMI_self − BMI_meas if 18.5 ≤ BMI_meas < 25	582	−0.42	0.71	−3.38	2.13	408	−0.18	0.81	−3.27	3.19
BMI_self − BMI_meas if 25 ≤ BMI_meas < 30	396	−0.79	0.92	−3.79	4.06	670	−0.54	0.90	−3.66	2.81
BMI_self − BMI_meas if BMI_meas ≥ 30	277	−1.06	1.09	−4.14	1.51	215	−1.02	0.92	−3.55	1.31
Obesity (BMI ≥ 30)										
Obese (measured)	1,266	0.22	0.41	0	1	1,294	0.17	0.37	0	1
Obese (self-reported)	1,266	0.18	0.39	0	1	1,294	0.13	0.33	0	1
Waist circumference										
Wasit circumference (centimeters)	1,266	84.92	13.32	56	164	1,294	94.55	9.87	63	145
Central obesity	1,266	0.35	0.48	0	1	1,294	0.19	0.39	0	1
Education, income and control variables										
Educ1 (<11 years)	1,266	0.27	0.44	0	1	1,294	0.29	0.45	0	1
Educ2 (two or 3 years of high school)	1,266	0.47	0.50	0	1	1,294	0.51	0.50	0	1
Educ3 (<3 years of post-secondary education)	1,266	0.15	0.36	0	1	1,294	0.12	0.33	0	1
Educ4 (≥3 years of post-secondary education)	1,266	0.11	0.31	0	1	1,294	0.07	0.26	0	1
Annual disposable income (SEK)	1,266	128,078	42,616	42,514	282,392	1,294	137,039	45,820	42,512	341,343
Age	1,266	46.69	11.28	30	76	1,294	46.95	11.58	30	76
Unmarried	1,266	0.09	0.28	0	1	1,294	0.14	0.35	0	1
Divorced	1,266	0.06	0.25	0	1	1,294	0.04	0.20	0	1
Widow	1,266	0.04	0.19	0	1	1,294	0.01	0.08	0	1
First generation immigrant	1,266	0.08	0.27	0	1	1,294	0.07	0.25	0	1
Second generation immigrant	1,266	0.05	0.22	0	1	1,294	0.04	0.19	0	1

Defining obesity as BMI ≥ 30, obesity prevalence increases by four percentage points for both men and women when using measured values instead of self-reports. Notably, when defining obesity using waist circumference instead, prevalence increases to 35 % among women. The increase is less pronounced among men.

**Fig. 1:**
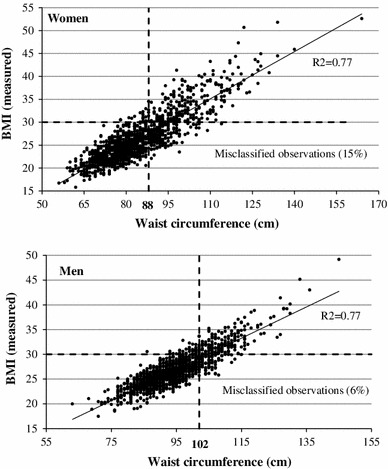
Relationship between BMI (calculated from measured height and weight) and waist circumference

Regarding misclassification, Fig. 1 illustrates the relationship between BMI (calculated from objectively measured height and weight) and waist circumference. Observations that are inconsistently classified in the sense that they are classified as obese according to one of the definitions but not according to the other are located in the upper left and lower right squares of each graph. In this study we focus on the lower right squares and define misclassification as being obese according to the waist circumference definition but not according to the BMI definition. 15 % of female observations, and 6 % of male observations, are misclassified in this sense.

Fewer men (3 %) and women (2 %) are allocated to the upper left corners, and thus have a relatively high BMI but a slim waistline. Clearly, these observations also represent a type of misclassification, namely being classified as obese according to the BMI definition, but not according to the waist circumference definition. However, the small number of individuals belonging to this type of misclassification makes it difficult to perform a comprehensive analysis of the determinants, and the misclassification analysis in this study therefore focuses only on the first type of misclassification.^6^

### Misreporting analysis

For the misreporting analysis, Tables 2 and 3 report the results from three different models. Model I includes three indicator variables for level of education in the ***x***_***i***_ vector. Model II includes the log of current household disposable income per consumption unit, and model III combines education and income. The first column reports the results from Eq. 1, where measured weight and height are used to calculate BMI to estimate “true” gradients. The second column contains the socioeconomic disparities based on self-reported information (Eq. 2). The third column shows the results from estimation of Eq. 3 and whether the estimates based on the self-reported data in column 2 are biased. The fourth column shows the results from estimation of Eq. 4 and whether there is any direct effect of socioeconomic status on the total bias.

**Table 2:** Misreporting analysis. Women

Dependent variable	BMI_meas	BMI_self	BMI_self − BMI_meas	BMI_self − BMI_meas
	Eq. 1	Eq. 2	Eq. 3	Eq. 4
	(1)	(2)	(3)	(4)
Model I				
BMI (measured)				−0.045***
				(0.006)
Educ2	0.098	0.170	0.071	0.076
	(0.389)	(0.383)	(0.072)	(0.071)
Educ3	−1.544***	−1.335***	0.209**	0.139*
	(0.485)	(0.471)	(0.086)	(0.084)
Educ4	−1.683***	−1.393***	0.290***	0.214**
	(0.470)	(0.457)	(0.090)	(0.087)
R-squared	0.11	0.094	0.106	0.165
Model II				
BMI (measured)				−0.046***
				(0.006)
ln (income)	−0.996**	−0.866*	0.130	0.084
	(0.465)	(0.454)	(0.083)	(0.081)
R-squared	0.094	0.081	0.098	0.162
Model III				
BMI (measured)				−0.045***
				(0.006)
Educ2	0.138	0.205	0.067	0.073
	(0.393)	(0.386)	(0.072)	(0.071)
Educ3	−1.476***	−1.275***	0.201**	0.134
	(0.486)	(0.474)	(0.086)	(0.084)
Educ4	−1.540***	−1.266***	0.274***	0.204**
	(0.483)	(0.471)	(0.092)	(0.090)
ln (income)	−0.600	−0.530	0.070	0.043
	(0.473)	(0.464)	(0.084)	(0.083)
R-squared	0.111	0.095	0.106	0.165

Robust standard errors in parenthesis. All regressions include a constant and controls for age, civil status, and immigration background. No. of observations: 1,266

*** *p* < 0.01, ** *p* < 0.05, * *p* < 0.1

**Table 3:** Misreporting analysis. Men

Dependent variable	BMI_meas	BMI_self	BMI_self − BMI_meas	BMI_self − BMI_meas
	Eq. 1	Eq. 2	Eq. 3	Eq. 4
	(1)	(2)	(3)	(4)
Model I				
BMI (measured)				−0.075***
				(0.008)
Educ2	−0.317	−0.335	−0.017	−0.041
	(0.255)	(0.244)	(0.066)	(0.063)
Educ3	−0.605*	−0.602*	0.003	−0.043
	(0.333)	(0.321)	(0.089)	(0.087)
Educ4	−0.925**	−1.048***	−0.123	−0.192**
	(0.382)	(0.365)	(0.096)	(0.092)
R-squared	0.066	0.062	0.071	0.150
Model II				
BMI (measured)				−0.074***
				(0.008)
ln (income)	0.136	0.067	−0.070	−0.060
	(0.325)	(0.308)	(0.088)	(0.083)
R-squared	0.061	0.056	0.071	0.148
Model III				
BMI (measured)				−0.075***
				(0.008)
Educ2	−0.334	−0.348	−0.014	−0.039
	(0.256)	(0.245)	(0.066)	(0.063)
Educ3	−0.642*	−0.633*	0.009	−0.039
	(0.334)	(0.323)	(0.088)	(0.086)
Educ4	−1.004***	−1.113***	−0.109	−0.184**
	(0.388)	(0.370)	(0.096)	(0.092)
ln (income)	0.310	0.255	−0.055	−0.032
	(0.331)	(0.313)	(0.088)	(0.084)
R-squared	0.066	0.062	0.071	0.150

Robust standard errors in parenthesis. All regressions include a constant and controls for age, civil status, and immigration background. No. of observations: 1,294

*** *p* < 0.01, ** *p* < 0.05, * *p* < 0.1

For women (Table 2), the estimation of model I shows, as expected, that there are statistically significant educational disparities when using BMI calculated from both measured and self-reported weight and height.^7^ These estimates are statistically significantly larger (i.e. more negative), by about 15 %, than the ones estimated from self-reported data. Hence, the estimated gradient based on self-reported values is biased towards zero. According to column 4, there is a direct effect of education on the bias for the highest education group (*p* < 0.05). Given the same level of true BMI, women in the highest educational group report weight and height in a way that results in less underreporting of BMI compared to the lowest educational group. Because women with higher education also tend to have lower BMI than those with lower education, and because women with lower BMI underreport BMI to a lesser extent than women with higher BMI, there is also an indirect effect of education on the bias. Following Eq. 5, the indirect effect is a combination of *ρ* and ***γ*** ^meas^, which for the highest educational group is (−0.045)*(−1.683) = 0.076, corresponding to about 26 % of the total bias.

In model II, using BMI calculated from measured weight and height results in a negative income gradient (*p* < 0.05). Using self-reported information gives a somewhat smaller, and less significant (*p* < 0.10), effect, but the difference between the two estimates is not significant (*p* > 0.10, column 3).

Combining education and income in model III shows that education seems to be a stronger correlate with BMI than income. Once controlling for education, income is insignificant (*p* > 0.10), and the size of the coefficient is reduced compared to model II. The negative correlation between education and BMI, however, remains statistically significant, although the sizes of the education coefficients are somewhat smaller compared to model I. The bias towards zero in the education gradient when using self-reported data remains very similar, as in model I.

Table 3 reports the male results. According to model I, there is a difference in BMI across educational groups also among men, where men in the highest educational groups differ by having a lower BMI (*p* < 0.10). However, this difference is smaller than among women. Compared to a man with 11 years of schooling at most, a man in the highest educational group (*educ4*) has about a 0.9 index point lower BMI. The income gradients in models II and III are positive, but small and insignificant.^8^ Controlling for both income and education in model III gives results similar to those in model I. Hence, as for women, education appears as the strongest BMI-related socioeconomic variable in this sample, whereas income is less important.

Unlike the female results, there is no evidence of significant differences between the specifications with self-reported and measured information (column 3). However, column 4 shows a *negative*, and statistically significant (*p* < 0.05), direct effect for the highest educational group; given the *same level of true BMI*, men in the highest educational group tend to underreport BMI to a *larger* extent than those in the lowest educational group. Because men in higher educational groups tend to have lower BMI in general (column 1), and because men with higher BMI underreport more (column 4), the indirect effect is positive, and, consequently, the total bias in column 3 is smaller than the direct effect. The total bias (column 3) shows a tendency towards an overestimation of the education gradient (i.e. a more negative education effect) when using self-reported data. This means that, overall, men in the highest educational group underreport BMI to a *larger* extent than those in the lowest educational group, despite their lower BMI in general. The bias is not statistically significant though, and altogether male educational disparities in BMI calculated from self-reported weight and height seem to be less biased than corresponding female disparities.

### Misreporting: sensitivity analysis

The robustness of the results presented in Tables 2 and 3 is explored in various ways.^9^ The first robustness check aims at taking into account that the number of days between the first and second visit to the health care center is not the same for all observations. Although all participants were supposed to make their second visit to the health care center 14 days after the first visit, the number of days between the visits generally varies between 0 and 60 days, with some additional outlying observations. The median is the intended 14 days for both men and women.

As the time period between the visits increases, the risk that the observed difference between BMI calculated from self-reported and measured information is an actual weight difference, and not a misreport, increases (height reasonably does not change in the age groups included in the analysis). To deal with this issue, we repeat the analysis on a constrained sample, including only individuals with information on when the first and second visit took place and for who the number of days between the visits is 65 days at most. This reduces the sample by 137 female and 159 male observations. To explore whether the results from the main analysis remain when taking the different lengths of time between the visits into account, the number of days and its square are added as control variables in all regressions.^10^

Among women, the number of days between the visits is indeed related to misreporting such that underreporting increases with the number of days. Compared to the main analysis, the bias in the educational gradient is somewhat reduced, and the statistical significance decreases somewhat.^11^ For income, no differences to the main analyses are observed. Also among men, underreporting increases with the number of days between the visits, but the correlation is statistically insignificant. However, controlling for the number of days between visits in the misreporting regressions reduces the size and precision of the bias in the educational gradient also for men. The direct effect of *educ4*, which is significant at the 5 % level in the main analysis, loses its statistical significance. Overall, the qualitative conclusions based on this constrained sample analysis are the same as for the main analysis, but the results are somewhat weakened in terms of size and statistical significance once controlling for the number of days between visits and removing observations with long periods between them. This could indicate that part of the misreporting are actual weight changes rather than misreporting, but also when accounting for this, there is evidence of bias in female educational gradients based on self-reported data.

Another concern could be that, with time, participants learned that they would first be asked about their weight and height, which would be measured later. Hence, because of learning, individuals who were surveyed and examined towards of the end of the period could misreport to a lesser extent than those examined in the beginning. To explore this possibility, the second repeated analysis includes control for the within sex and municipality rank for when the second visit at the health care center occurred, and the square of this variable.^12^ There is no apparent tendency that individuals who were examined towards the end of the period reported more accurately and the rank variables are not statistically significantly related to misreporting. Overall, the results from this alternative analysis are very similar to the results from the main analysis.

In a third round of robustness checks, the sample is repeatedly modified by excluding certain groups, with the purpose of ensuring that the results in the main analysis are not driven by a particular subgroup, and that results are not too sensitive to removal of these subgroups. Different age groups (74–76, 70–76, 65–69, 60–64, 55–59, …, 30–34), the different civil status groups (unmarried, divorced, and widow), and the immigration status groups (first and second generations) are removed, one by one, resulting in 15 repetitions of the analysis.

Among women, the bias in the estimate of *educ3* when using self-reported BMI is statistically significant at the 5 % level in 12 out of the 15 repeated analyses, the bias in the estimate of *educ4* is significant (with *p* < 0.05 or better) in all samples, and the direct effect of *educ4* goes from being significant at the five percent level to being significant at the ten percent level in three cases.^13^ Among men, the difference between estimates based on self-reported and measured BMI remains negative but statistically insignificant (*p* > 0.10) in all 15 samples. The direct effect of *educ4* in the main analysis remains negative in all samples, and significant (with *p* < 0.05 or better) in half of the samples.^14^ Overall, we conclude from this third part of the sensitivity analysis that the exact estimates vary somewhat across samples. Nevertheless, qualitatively, the key patterns from the main analysis are identified also in the majority of the subsamples explored. It is difficult to reveal any clear patterns for when the results change compared to the main analysis. The most consistent result throughout is that the results weaken when the age group 70–76 is removed, indicating that the biases observed in the main analysis might be stronger in this age group.

### Misclassification analysis

Turning to the misclassification analysis, Table 4 reports the results from estimation of Eq. 6. Among women, the tendency of being misclassified goes in opposite directions for education and income. The probability of being misclassified decreases with income, whereas it is (insignificantly) larger for women in the highest educational group. Because education and income are positively correlated, including only one of the variables also captures the correlation with the other. Consequently, including both education and income in model III strengthens the correlation with both income and education compared to model I and model II, respectively. According to model III, a 10 % higher income is related to a 0.75 percentage point decrease in the probability of being misclassified. The education variables remain positive but insignificant.

**Table 4:** Misclassification analysis

	Dependent variable: misclassification A					
	Women (*n* = 1,266)			Men (*n* = 1,294)		
	Mean of dependent variable: 0.150			Mean of dependent variable: 0.057		
	(1)	(2)	(3)	(4)	(5)	(6)
	Model I	Model II	Model III	Model I	Model II	Model III
Educ2	0.000		0.005	−0.021		−0.019
	(0.028)		(0.029)	(0.018)		(0.019)
Educ3	−0.003		0.005	−0.035		−0.029
	(0.036)		(0.037)	(0.021)		(0.022)
Educ4	0.038		0.056	−0.049**		−0.037
	(0.041)		(0.042)	(0.024)		(0.025)
ln (income)		−0.064*	−0.075**		−0.052**	–0.045**
		(0.035)	(0.036)		(0.021)	(0.022)
R-squared	0.060	0.062	0.064	0.056	0.058	0.060

Linear probability models. *** *p* < 0.01; ** *p* < 0.05; * p < 0.1. Robust standard errors in parentheses. All regressions include a constant and controls for age, civil status, and immigration background

Among men, there is a negative correlation between misclassification and both the highest educational group and income. In model I, men in the highest educational group are 4.9 percentage points less likely to be misclassified than those in the lowest educational group. In model II, a 10 % higher income implies a 0.52 percentage points lower probability in being misclassified. When controlling for both income and education in model III, the association with income remains statistically significant (*p* < 0.05) whereas education loses its significance.

Systematic variation across income and education in misclassification implies that income and education gradients differ depending on the definition of obesity. Table 5 illustrates this implication by reporting the results from estimating Eq. 7, where obesity is defined in three different ways. For women, columns 1–2 show that educational differences increase when moving from BMI calculated from self-reports to BMI calculated from measured information to define obesity. This observation is in line with the misreporting analysis and the finding of a bias in the female educational gradient when using self-reported data on weight and height to calculate BMI (Table 2). When moving further, to obesity defined by waist circumference (column 3), the *educ3* estimate remains similar in size. The *educ4* estimate reduces in size, from around 13 percentage points to 5–8 percentage points, and becomes insignificant. This result reflects what Table 4 shows; women in the highest educational group tend to be misclassified more often, and hence the difference in obesity across educational groups depends on what definition is used. Similarly, because misclassification decreases with income (Table 4), the income gradient is significantly larger (*p* < 0.10 in model II and *p* < 0.05 in model III) for the waist circumference definition.

**Table 5:** Estimation of socioeconomic disparities in the risk of obesity for different definitions of obesity

Dependent variable	Women (*n* = 1,266)			Men (*n* = 1,294)		
	BMI ≥ 30 self-reported	BMI ≥ 30 measured	Waist circum-ference >88 cm	BMI ≥ 30 self-reported	BMI ≥ 30 measured	Waist circum-ference >102 cm
Mean	0.183	0.219	0.349	0.127	0.166	0.191
	(1)	(2)	(3)	(4)	(5)	(6)
Model I						
Educ2	0.041	0.019	0.014	−0.014	−0.012	−0.022
	(0.031)	(0.033)	(0.037)	(0.024)	(0.027)	(0.029)
Educ3	−0.069**	−0.096**	−0.103**	−0.022	−0.015	−0.044
	(0.035)	(0.038)	(0.045)	(0.033)	(0.038)	(0.038)
Educ4	−0.092***	−0.135*** (a**)	−0.080	−0.051	−0.051	−0.082**
	(0.035)	(0.037)	(0.050)	(0.036)	(0.041)	(0.042)
R-squared	0.067	0.096	0.097	0.051	0.049	0.057
Model II						
ln (income)	−0.068*	−0.060	−0.127*** (b*)	−0.022	0.020 (a*)	−0.031 (b*)
	(0.035)	(0.038)	(0.045)	(0.033)	(0.037)	(0.038)
R-squared	0.054	0.081	0.095	0.050	0.048	0.055
Model III						
Educ2	0.044	0.021	0.021	−0.013	−0.013	−0.021
	(0.031)	(0.034)	(0.037)	(0.024)	(0.027)	(0.029)
Educ3	−0.064*	−0.093**	−0.090**	−0.020	−0.019	−0.042
	(0.035)	(0.038)	(0.046)	(0.033)	(0.037)	(0.038)
Educ4	−0.081**	−0.128*** (a**)	−0.054 (b*)	−0.048	−0.058	−0.078*
	(0.036)	(0.038)	(0.050)	(0.037)	(0.042)	(0.043)
ln (income)	−0.045	−0.028	−0.109** (b**)	−0.014	0.029	−0.018
	(0.037)	(0.038)	(0.046)	(0.033)	(0.037)	(0.039)
R-squared	0.068	0.097	0.102	0.052	0.050	0.058

Linear probability models. Robust standard errors in parentheses. All regressions include a constant and controls for age, civil status, and immigration background. (a): the estimate is statistically significantly (asterisks referring to significance level) different from the corresponding estimate where BMI ≥ 30 calculated from self-reported weight and height are used to define obesity (column 1 for women and column 4 for men). (b): the estimate is statistically significantly (asterisks referring to significance level) different from corresponding estimate where BMI ≥ 30 calculated from measured weight and height is used to define obesity (column 2 for women and column 5 for men)

*** *p* < 0.01, ** *p* < 0.05, * *p* < 0.1

For men (columns 4–6) the coefficients of the education variables are basically the same for obesity defined by self-reported and measured BMI, which is in line with the results from the misreporting analysis (Table 3) where no bias in the education disparities was detected. Notably, using self-reported or measured BMI to define obesity, there are no statistically significant differences in obesity across education or income. However, when using waist circumference to define obesity, a negative education effect for the highest educational groups evolves. The size of this difference is about 8 percentage points (*p* < 0.05 in model I and *p* < 0.1 in model III). There is also a negative income gradient emerging, reflecting the result from the misreporting analysis that the probability of misclassification increases with income. However, the income coefficient is rather small and does not reach statistical significance.

### Misclassification: sensitivity analysis

As for the misreporting analysis, the robustness of the results from the misclassification analysis is checked by repeating the analysis on modified samples where age groups, civil status groups and immigration groups are removed, one at a time, to ensure that the results in the main analysis are not driven by a particular subgroup.^15^^,^ 16 Among women, the tendency for those in the highest educational group to be misclassified more often remains in all repeated analyses. The association also remains statistically insignificant (*p* > 0.05) in all cases but one.^17^ The negative correlation between misclassification and income remains negative in all 15 subsamples. Controlling for education (model III), it varies between −0.051 and −0.098. In terms of statistical significance, the clearest pattern is that the significance disappears (*p* > 0.10) when the four youngest age groups are removed (i.e. 30–34, 35–39, 40–44, and 45–49).^18^ This result could imply that the negative correlation between income and misclassification is more apparent among those under the age of 50, but it could also be a result of smaller sample sizes.^19^

Among men, those in the highest educational group are less likely to be misclassified in all 15 subsamples, but the difference compared with those in the lowest educational group is statistically significant (*p* < 0.05) in six cases only. Controlling for both education and income (model III) results in a rather stable association between income and misclassification, varying between −0.045 and −0.055, which is significant (*p* < 0.05) in nine out of the 15 subsamples.^20^ Hence, as in the main analysis, the correlation between income and misclassification appears more stable and pronounced than the lower probability of those in the highest educational group to be misclassified. There is no clear indication as to whether this overall result from the main analysis would be sensitive to exclusion of any particular age, civil status or immigration status group.

The definition of obesity based on waist circumference takes only waist circumference, and not height, into account, whereas BMI is weight relative to height. There might therefore be a concern that the misclassification is related to height. The height variables are indeed jointly significantly related to misclassification such that the probability of being misclassified increases with height. However, adding controls for height does not have any major impact on the relationship between misclassification and education or income. The negative association between income and misclassification for women remains significant (with *p* < 0.05), and becomes stronger rather than weaker, when controlling for height. Among men, the negative association between misclassification and both income and education strengthens somewhat once controlling for height. Along the same lines, the emerging educational gradient among men which Table 5 observed remains also when height is controlled for in the obesity probability regressions, and it becomes stronger rather than weaker.

A final set of robustness checks regards the estimation method used in the misclassification analysis. The main analysis relies on linear probability models (LPM). A major and well-known shortcoming with the LPM is that it may well predict probabilities outside the unit interval [33]. The non-linear probit and logit models are alternatives that eliminate this problem. As a robustness check, we therefore perform the misclassification analysis using probit and logit models, calculating average marginal effects as well as marginal effect at the mean. Overall, results are not particularly sensitive to estimation method. Among women, results are very similar irrespective of estimation method. Among men, results are also similar, although standard errors are somewhat smaller when using the non-linear models, resulting in somewhat improved significance levels.

The overall conclusion from the misclassification sensitivity analysis is that the results from the main analysis are robust to estimation method (i.e. whether LPM, logit or probit is used) and to whether height is included as a regressor or not. However, the exact estimates vary somewhat when different subgroups are removed, and results are somewhat sensitive to the exact sample. But it is difficult to reveal any clear patterns of when the results change compared to the main analysis.

## Discussion

This study deals with the potential problems related to, first, relying on self-reported weight and height to calculate BMI (*misreporting*), and, second, the concern that BMI is a deficient measure of body fat and elevated health risks (*misclassification*). We analyze how these potential problems affect estimates of socioeconomic disparities in BMI and obesity, where education and income are used as measures of socioeconomic status. Although misreporting and misclassification are acknowledged in the literature, and although misreporting by socioeconomic status has been studied previously, to our knowledge, there is no previous evidence on how estimated educational and income disparities in BMI and obesity are affected by the misreporting and misclassification. Previous studies do not generally discuss or analyze how and whether self-reports and misclassification bias estimated socioeconomic disparities [4, 6, 7]. For occupation, Boström and Diderichsen [5] conclude that misreporting results in underestimated disparities in the risk of obesity among women, and in overestimated disparities in overweight and obesity among men. Regarding misclassification, Burkhauser and Cawley [1] briefly note that the negative correlation between employment and obesity increases for men, but not for women, when using an alternative measure of obesity. Our study focuses explicitly on the issue of how socioeconomic disparities are affected by misreporting and misclassification. For the misreporting analysis, we develop a modeling framework that distinguishes between a direct and an indirect effect of socioeconomic status on misreporting.

In the misreporting part we find that women with higher education misreport less than those with lower education, which is in line with findings in Dekkers et al. [6] and Nyholm et al. [4]. The consequence of this systematic difference in misreporting across education is that when analyzing educational disparities in BMI derived from self-reported weight and height, the resulting disparities are underestimated (i.e. biased towards zero), compared to disparities derived from objectively measured data. The bias is a combination of a direct and an indirect effect of education. The direct effect means that women in the highest educational group misreport less *given the same BMI*. The indirect effect stems from BMI being related to misreporting in combination with a relationship between education and BMI per se. We find no strong evidence of a similar bias in income disparities. Among men, there is a direct effect of education on misreporting such that for a given level of given BMI, men in the highest educational group misreport to a *larger* extent than those in lower educational groups. However, because men in the highest educational group tend to have lower BMI, and BMI is related to misreporting, the indirect effect counteracts the direct effect, and in total we find no statistically significant bias in the education disparity based on self-reported weight and height to calculate BMI. We find no significant income disparity in BMI among men, or any evidence of a bias in it.

In the misclassification part we use waist circumference as an alternative definition of obesity beyond the commonly applied definition based on BMI. We find that the probability of being misclassified, defined in this study as being obese according to the waist circumference definition but not according to the BMI definition, decreases with income, for both men and women. As a result, among women we find a somewhat steeper income gradient in obesity when using waist circumference instead of BMI as the basis of definition. Among men, the income gradient is statistically insignificant irrespective of how obesity is defined. We also find a tendency that men in the higher educational groups are misclassified less often, although statistically insignificantly so. Nevertheless, when estimating the probability of obesity defined using waist circumference, an educational gradient, which is not present when classifying men using BMI, arises. Among women, the difference in obesity rates between the highest and lowest educational groups is reduced when using waist circumference to define obesity.

The robustness checks show that results may differ somewhat depending on the exact sample used in a way that could be interpreted as pointing towards differing biases in socioeconomic disparities across age groups. This is a topic that could be further analyzed in a future study.

The reason behind the stronger female income differences and emerging male educational differences when using waist circumference to define obesity is not explored in the study. To explore it properly and carefully one would need to learn more about the determinants of abdominal obesity, as opposed to obesity defined based on BMI, and factors determining where the body stores body fat. One speculative and potential explanation is that various lifestyle behaviors, like physical activity and diets, differ by socioeconomic status [34] and that these differences could result in different productions of abdominal fat, resulting in a slimmer waist line but not necessarily in lower weights to the same extent. However, again, more needs to be learnt about which factors affect fat storage, and no strong conclusions about the reasons should be drawn from the results of this study.

It is important to note that this study deals with differences in BMI and obesity between educational groups and across the income distribution, but does not establish causal relationships. Hence, we do not claim that a higher education or a higher income *causes* a lower BMI or a lower probability of obesity. Along the same lines, we do not claim that a higher education among women *causes* these women to report weight and height in a way that results in more accurate BMI. Rather, we observe systematic differences in misreporting and misclassification across different groups and conclude that these differences matter for the estimation of socioeconomic disparities in BMI and obesity. Although no causality in any relationships should be inferred from this study, it is illuminating to see how correlations between socioeconomic factors like education and income on the one hand, and obesity and BMI on the other hand, are biased because of misreporting and misclassification.

The overall conclusion from the study is that misreporting and misclassification may indeed matter for estimated educational and income disparities in BMI and obesity. If the results in this study are assumed to also hold for other populations, previous and future results about socioeconomic disparities based on self-reported BMI are likely to underestimate the true educational differences among women. Likewise, female educational gradients in obesity are biased towards zero if self-reported BMI is used to define obesity, instead of measured BMI or waist circumference. Female income gradients in obesity will be smaller if BMI is used to define obesity than if waist circumference is used as the underlying measure. For men, income and educational disparities in BMI, and obesity defined using BMI, do not seem to be sensitive to whether BMI is calculated from self-reported or measured weight and height. However, there might be a male educational disparity in abdominal obesity which is being overlooked when using BMI to define obesity. In short, female educational disparities in BMI and obesity appear sensitive to whether self-reported or measured weight and height are used, which results in an underestimation of the disparities. Moreover, for both men and women, some attention should be paid to the definition of obesity, as different definitions seem to give somewhat different results in terms of educational and income disparities.

Similar to studies using validation data to correct measurement errors, as well as to other studies using regional data sets, the generalizability of the results of this study is limited by the characteristics of the data set. For the results of the misreporting analysis in this study to be useful in a broader context, for example in order to draw conclusions at the national level or for studies on other populations, the distribution of BMI calculated from measured height and weight, conditional on the distribution of the corresponding self-reported values, socioeconomic status, and other variables included in the analysis must be the same in both populations. Likewise, for the misclassification results to be valuable in a broader context, the inter-relationships of central obesity, BMI, age and socioeconomic status must be similar in both contexts. It is difficult to judge whether these conditions are likely to hold. The sample used in the analysis is possibly a selected one, which is not representative for the region or for the whole country. In the region where the data for this study were collected, the fraction of individuals with at least 3 years of post-secondary education is somewhat smaller, and the fraction with low education is somewhat larger, compared to the average for Sweden [35]. Moreover, according to a report comparing health outcomes across Swedish municipalities and based on survey data collected between 2006 and 2008, obesity prevalence (defined as BMI ≥ 30 calculated from self-reports) among men and women aged 18–80 is somewhat higher in the region under consideration in this study than the average for the country as a whole, although confidence intervals overlap [36]. However, these factors do not necessarily imply that misreporting behavior and misclassification patterns are different to the rest of Sweden, or to any other population. Overall, despite the regional character and the generalization limitation, and without being able to ensure that results would be the same in other samples, we believe that our results add valuable insights into the nature and consequences of misreporting of BMI and misclassification of obesity. Similar analyses using other data will be useful complements to this study.

## Footnotes

1 The sample consists of individuals aged 30–76, see Section “Data, variables and sample”.

2 It may be useful to note that Eq. 4 can be re-written as BMI_self_*i*_ =α_4_ + (1 + *ρ*)*BMI_meas_*i*_ + ***z***_***i***_ *** ***β*** ^direct^ *+* ***x***_***i***_ *** ***γ*** ^direct^ *+* *r*_*i*_, i.e. without BMI*_*meas on both the left and right hand side. *β* and *γ* will have the same estimated values irrespective of which equation that is used in the empirical implementation.

3 Equations 1–5 could also be specified with the BMI variables transformed into their log values, producing a non-linear approach to the relationship between measured BMI and the size of misreporting. The interpretation of ln(BMI_meas) in Eq. 4 would be that a 1 % higher measured BMI implies a *ρ* change in the percentage difference between self-reported and measured BMI. Hence, given *ρ* < 0, this model results in larger absolute misreporting for higher levels of measured BMI, which may be a plausible feature. The interpretation of the education and income (and other) variables change as well. Because interpretation differs, results cannot be compared straight away. However, at sample means, the results from the log transformation and from the level models are similar. Because interpretation is considerably more straightforward when the BMI variables are in levels, this version is the one presented and used in the study. Results from the log versions are available from the authors upon request.

4 There is no information in the data about individuals who were invited to participate but chose not to do so. Likewise, there is no information about individuals who participated in the first visit but not in the second, and hence never got their weight, height and waist circumference measured. Consequently, we cannot check selection and attrition issues, i.e. whether individuals who chose to participate were different from those who did not want to participate, or if individuals who chose to complete the survey by going to the second visit were different from those who chose to stop after the first visit. Hence, the sample used in the analysis, and the results based on it, hold for the specific final sample used, but are possibly not representative for the full population in the region.

5 Income is provided for the year when the individual participated in the health survey (i.e. 2001, 2002, 2003, 2004 or 2005). Incomes for years before 2005 are adjusted for inflation by the consumer price index. Different household members have different consumption weights depending on age and household size and composition. The consumption weights are as follows [32]: the first adult in the household has 1.16, the second co-habiting adult has 0.76, others above 18 years of age have 0.96, children 11–17 years old have 0.76, children 4–10 years old have 0.66, and children 0–3 years old have 0.56.

6 We have analyzed both types of misclassifications, but do not find any results of importance or interest for the second type of misclassification, possibly because of the small number of misclassified individuals of this type.

7 To exemplify the interpretation of the educ variables, based on measured information (column 1), BMI among women in the two highest educational groups are about 1.5 and 1.7 index points lower, respectively, compared to women in the lowest educated group.

8 The interpretation of the coefficient of ln(income) is that a 1 % increase in income is related to a β/100 difference in BMI. Hence, for example, in model II, a 100 % higher income is related to a 0.13 index points higher BMI, which is a rather small difference compared to the association between education and BMI in model I.

9 Detailed results from all sensitivity analyses are available from the authors upon request.

10 The square version of the variable is added in order to allow for a non-linear relationship between the time between the visits and the size of the misreporting. However, the results show no such strong tendencies.

11 In both models I and III, the significance level of the total bias in the educ4 estimate reduces from the 1 to the 5 % level when accounting for the number of days between visits. The direct effect of educ4 changes from significance at the 5 to the 10 % level.

12 The square version of the variable is added in order to allow for a non-linear relationship between the rank and the size of the misreporting.

13 The three samples when the bias in *educ3*, and the direct effect of *educ4*, goes from being significant with *p* < 0.05 to *p* < 0.10 are when age groups 70–76 and 55–59, and when the unmarried, are removed.

14 The eight samples where the significance level drops to *p* > 0.05 are when the following groups are removed: age 74–76, age 70–76, age 65–69, age 45–49, age 40–45, age 35–39, unmarried, and first generation immigrants.

15 Unlike the misreporting analysis, the variation in the number of days and the order in which the visits took place are not concerns for the misclassification analysis because misclassification is based on information from the second visit only.

16 As for misreporting, the full results from the sensitivity analysis are available from the authors upon request.

17 In model III, when the age group 30–34 is excluded, the positive association between *educ4* and misclassification is significant (*p* < 0.05).

18 In addition, the significance level reduces to *p* < 0.10 when the following three groups are removed (one at a time): age 60–64, age 55–59, and second generation immigrants.

19 These four groups are the four largest groups explored in this sensitivity analysis, with number of observations between 328 and 531. The number of observations in the other age, civil status and immigrations groups varies between 56 and 295.

20 The income coefficient loses statistical significance (*p* > 0.05) when the following six groups are removed (one at a time): age 30–34, age 45–49, age 50–54, divorced, single (as opposed to being married or cohabiting), and first generation immigrants.
